# Creating a meta-REM map: pragmatic improvements to ripple effects mapping methodology used to support evaluations of complex public health systems

**DOI:** 10.1186/s12874-026-02811-6

**Published:** 2026-02-24

**Authors:** Louise Padgett, Philip Garnett, Maria Bryant, Laura Nixon, Patience Gansallo, Amy Creaser, Bridget Lockyer, Jessica Sheringham, Liina Mansukoski

**Affiliations:** 1 Department of Health Sciences, University of York, York, England, UK; 2 School of Business and Society, University of York, York, England, UK; 3 Department of Health Sciences, Hull York Medical School, University of York, York, England, UK; 4 Wolfson Institute of Population Health, Queen Mary University of London, London, England, UK; 5 Public Health and Prevention Department, Doncaster City Council, Doncaster, England, UK; 6 Leeds Institute of Health Sciences, University of Leeds, Clarendon Way, Leeds, LS2 9NL England, UK; 7 Bradford Institute for Health Research, Bradford Teaching Hospitals NHS Foundation Trust, Bradford, England, UK; 8 Department of Primary Care and Population Health, University College London, London, England, UK; 9 Hull York Medical School, University of York, York, UK

**Keywords:** Ripple effects mapping, Systems evaluation, Public health programme

## Abstract

**Introduction:**

Ripple Effects Mapping (REM) is a qualitative method that supports the evaluation of complex interventions within a system through mapping events that lead to intended and unintended consequences. Ongoing development of REM methodology is recommended to meet the needs of different evaluations. This paper outlines advancements to the REM method to facilitate the creation of a ‘meta-REM’ – a consolidated REM output that verifies and incorporates information from multiple REM workshops. We used a complex, public health programme, ‘ActEarly’, as a case study.

**Methods:**

Data collection involved workshops with key stakeholders who mapped activities along a timeline to show how they interlinked to create impact. Additional, novel processes were conducted to consolidate individual REM outputs (maps depicting ripples of activities) across multiple REM workshops to create an overall meta-REM map depicting overall activities within the programme. This was achieved through: 1) Extracting all activities (nodes) from each output 2) Identifying and consolidating duplicate nodes 3) Applying and verifying dates to nodes through triangulation with wider programme documentation 4) Inputting nodes with allocated dates into a spreadsheet 5) Inputting nodes according to the ripples in the original REM outputs within a separate spreadsheet within the same workbook 6) Using the software Graphviz to generate a meta-REM map from the data in the spreadsheet depicting all activities and ripples.

**Results:**

REM outputs from five workshops with different stakeholders were digitally combined to create a meta-REM map (n=173 nodes). This allowed the identification of the most influential activities within the ActEarly programme, and an understanding of how the type of activities changed over the programme’s life course. In addition, activities that impacted more than one study site were identified, as well as an those that led to multiple outputs or impacts.

**Conclusion:**

Meta-REM is useful for those conducting multiple REM workshops and evaluating large programmes across multiple localities in visualising and analysing data as a whole. This method can provide additional insights compared with individual analysis of REM outputs. A meta-REM map can facilitate deeper understanding of how a programme changes over its lifetime and the most influential activities causing impact creating further knowledge to support programme evaluation.

**Supplementary Information:**

The online version contains supplementary material available at 10.1186/s12874-026-02811-6.

## Background

Ripple Effects Mapping (REM) is increasingly used and adapted to evaluate the contribution of activities and interventions in public health research [1, 2]. It is a qualitative participatory evaluation method involving a variety of stakeholders who are directly and indirectly involved in the programme, and asked to visually map the chain of events within an intervention or programme to highlight both its contribution to intended and unintended consequences [1]. The idea is to encourage the stakeholders to evaluate programmes, interventions or collaborations, typically at their completion, by getting them to consider changes across groups, organisations and communities that took place due to the activity that is being evaluated [1]. Evaluations using REM can capture the impact of complex programmes [1] and can result in future recommendations around how best to implement similar interventions [3]. More recently, REM has been deemed particularly helpful in systems evaluation of public health research with specific adaptations to the method (i.e., the addition of a timeline) helping to account for the adaptive and complex nature of systems [2]. To date, REM methods have been applied in a number of different areas of health research, including community development [4], physical activity interventions [2, 5] and childhood obesity prevention [6, 7]. Previous evaluations have used REM to explore perceived causal pathways to outcomes [5, 7] of complex interventions at individual and community levels [6, 7].

As the popularity of the REM method has increased, novel challenges have emerged that require additional adjustments to the methodology, as well as solutions to analyse the resulting data. One key challenge is the building of a representative visualisation through stakeholder workshops. The approach as introduced by Chazdon et al., [1] provides an opportunity for the perspectives of multiple stakeholders to be brought together to document and map their understanding of the programme impact [1]. The result is a visualisation, which is intended to represent programmes at multiple levels and understand the context of the changes, and thus, the pathways through which activities and impacts. The mapping activities can aid reflection on experiences and increase depth of responses [4, 8]. In addition, where REM workshops are conducted at different time points within a programme, workshops can foster connections between stakeholders and enthusiasm for the programme [1]. Data collection methods within REM have also been deemed relatively straightforward [9] and cost-effective [4]. Reflections on REM methods by Creaser et al., [3] recommend that sufficient time is allowed to allow participants to provide as much information as possible. In addition, gaining a thorough understanding of who the REM participants are prior to workshops can ensure that workshops are engaging and meaningful [3].

REM can be used alongside other evaluation methods [4] to incorporate an understanding of context and its impact on REM outputs. Further, Nobles et al., [2] has proposed the inclusion of multiple workshops to run concurrently throughout the programme, as well as including a timeline within the mapping process to allow the maps to capture changes within a system over time and aid understanding of how long impacts take to occur [2]. A further advancement has since been the proposed merging of REM and realist methods [9]. This method, referred to as Realist Ripple Effects Mapping (RREM) provides an understanding of the mechanisms that explain how and why changes within the system occur over time [9]. Though very useful, the existing approaches to REM do not describe the practical steps that need to be taken to consolidate the collected information from multiple workshops into a map that is meaningful and ‘truthful’ (i.e., arrows indicating the passage of time only go forwards, event timeline (broadly) reflects reality, there is no duplication of events and ripples etc.).

How REM data is analysed varies dependent on the needs of the individual research project [3]. Frameworks such as the Community Capitals Framework [10] which considers the extent to which activities cause impacts and changes across the different capitals (i.e., human, social, political) are often used [11], as well as inductive analysis approaches [2], grounded theory [4] and ‘retroductive’ approaches that aim to explain causal connections underpinning pathways have been used [9]. Often wider data sources (i.e., from follow up interviews with participants or transcripts of the REM workshops) are used within analysis [4, 9, 12]. In addition, the level data is coded at can vary within REM methods. Some studies code “impact pathways” [2] or ripples [5], whereas others use individual nodes from the maps as a starting point and use wider data sources (i.e., interview data) to provide more understanding to data from the maps [12]. The advancements and variation in REM methods highlight the flexibility and varied use of the methodology [2]. However, what the existing analytical approaches do not specifically address is how to code the REM data (both for the purposes of the visualisations as well as analysis) and how to ‘sense check’ the resulting information. This is particularly pertinent in situations where there are multiple stakeholder groups involved in generating the REM.

The aim of this research was to develop and evaluate a process for consolidating REM outputs to facilitate data analysis and presentation. This paper tests the process through a case study and aims to demonstrate how a REM “meta-map” can be created, while assessing the strengths and challenges of this approach. This is intended to address challenges in analysing REM data from complex adaptive systems and of ‘meta-evaluations’ where the intervention or programme being evaluated is a large cluster of multiple related projects and activities where multiple REM outputs are created. We introduce pragmatic solutions using a case study approach, through the meta-evaluation of ActEarly, a complex, city-wide public health programme which aimed to improve the health and wellbeing of children and families [13, 14]. We show how additional processes following data collection can enable the generation of a meta-REM which depicts all impact pathways across the system according to the timeline of the programme. This approach enables the visualisation of impacts across the whole system, identifies key events leading to multiple pathways of impact and describes how the number and type of activities change over the lifetime of the programme.

## Methods

### Case study for meta-REM: ActEarly – a city collaboratory approach to early promotion of good health and wellbeing

The ActEarly programme was a research consortium of interdisciplinary expertise that aimed to improve the health and wellbeing of families and reduce related inequalities within two areas that have high levels of deprivation in the UK; Bradford (in West Yorkshire) and London Borough of Tower Hamlets (LBTH) [13]. The programme applied a city-wide, systems approach to implement system level change through co-producing and implementing upstream prevention solutions [13]. ActEarly comprised of five main themes including Healthy Places; Healthy Learning, Food and Healthy Weight, Play and Physical Activity and Healthy Livelihoods. The programme involved 68 interlinked projects, including some involving citizen science and coproduction with local communities. A further Evaluation theme evaluated the overall ActEarly programme. REM methods were used as part of a range of other research methods, within a meta-evaluation (an evaluation of evaluations) of the programme as a whole [13]. This meta-evaluation aimed to understand the programmes impact on early life health and wellbeing outcomes, research collaboration and capacity building and local and national decision making [13]. Ethical approval was gained from University College London Research Ethics Committee (reference number: 2037/004).

### Ripple effects mapping

The underpinning principles and processes for conducting REM workshops aligned with those outlined in methods reported by Chazdon, Emery [1] and Nobles, Wheeler [2] (see Supplementary File 1). In the ActEarly case study, 22 participants took part in one of five workshops conducted between August and October 2023. Additional steps (i.e. verifying of timelines of activities, creating an overall meta-REM map and interpretation of the meta-REM map) were conducted within the analysis process to digitally merge REM outputs and create a meta-map. Table 1 summarises the REM processes followed within the ActEarly metaevaluation, which previous methods were drawn on and the adaptations made to meet the needs of this project.

**Table 1:** Meta-REM checklist

	Original REM Method (Chazden et al., 2017)	Adapted REM Method (Nobles et al.)	Additional processes to create a meta-REM map (based on ActEarly meta-evaluation)
*Application of REM principles* (appreciative inquiry, participatory approach, group interviewing, mind mapping)	✓	✓	✓
*REM workshops *(Introduction and overview, team or group-based discussions, mapping, reflecting on the impacts)	✓	✓	✓
*Inclusion of a timeline during mind mapping* (Participants report activities and ripples along the timeline of the programme)	✓	✓	✓
*Follow-up REM workshops* (follow up with the same participants at multiple time points)		✓	Optional
*Verification of timelines within REM workshop outputs* (application of specific dates to activities within outputs using wider programme data sources)			✓
*Digitalising of REM outputs* (Handwritten REM outputs are turned into digital versions)	✓	✓	Optional
*Merging of REM outputs *(Individual outputs from each workshop are combined using systems mapping software to present an overall meta- REM map)			✓
*Analysis of REM outputs* (data is coded and analysed deductively using an existing framework or inductively and developed into themes)	✓	✓	✓
*Potential extra step of overall analysis* (identification of most impactful activities, cross over ripples, change in activities over time)			✓

### Recruitment

Participants were researchers, contract researchers, ActEarly research theme leads, co-investigators and principal investigators. These participants were purposively selected based on methods proposed by Chazdon et al., [1] that both direct (e.g., implementation staff; ActEarly team members) and indirect (i.e., those influenced as a by-product of the intervention; ActEarly partner members) individuals can contribute to understanding pathways to impact.

### Data collection – REM workshops

Three workshops took place in person and two were conducted using the online conferencing platform, Zoom. Two workshops included participants from the Bradford site only, one included participants from the LBTH site only and two workshops included participants from both research sites. The ActEarly case study also included the added element of a timeline detailed by Nobles, Wheeler [2]. A total of 22 ActEarly stakeholders (including theme leads, research fellows, project managers, local government partners and co-directors) took part in one of the five workshops which took place towards the end of the project between August and October 2023.

The purpose of the workshops was to create ripple effects maps of ActEarly activities and interventions along a timeline (from the programme start date to present) highlighting how they interlinked to generate impact. Participants were encouraged to consider some of the key outcomes of ActEarly and then consider the relationship between stakeholders, ActEarly activities and interventions in the system and how they interact to impact the outcomes. Arrows were drawn between the events to illustrate the ripple effect. In person workshops involved participants directly drawing the map, whereas within online workshops, facilitators digitally drew the maps according to the discussions of participants using the presentation software, Miroboard.

### Analysis

The analysis processes included the stages of extracting data from individual REM outputs, digitising each output and coding. A deductive analysis approach was followed where activities within REM outputs were coded according to the programme logic model. Nodes that could not be coded to the logic model were recorded separately and supported interpreting data. The proposed additional processes of verifying dates of each activity and merging individual REM outputs were also conducted.

### Data verification

Nodes (individual activities) were extracted from each workshop output onto a Microsoft Excel spreadsheet. Each node was provided a full label (i.e. full project titles and job roles or abbreviations spelled out in full) and where necessary clarification of node names was sought through member checking with participants or through confirmation with relevant ActEarly documents (e.g. ActEarly project log). Duplicate nodes were identified across the different workshop outputs to aid with consolidating outputs into one meta-map (i.e., ensuring they all have the same label and date). Unlike in previous approaches [1, 2], where possible, dates were assigned to nodes to depict when the activity happened or started. Date information was sought from REM workshop participants, wider ActEarly members or through relevant ActEarly documentation (i.e., ActEarly website, publications, project logs or data collected to support documentary analysis). Nodes that were more conceptual and could therefore not be dated (e.g. relationship building) were reported separately to be drawn upon to aid interpreting REM outputs. Nodes that could not be assigned dates were not included in the meta-map. Ensuring the accuracy of dates of each activity was important so that where the same activity was reported across multiple outputs, each ripple would sync up. Additionally, the verification of dates could provide more specific insights into the time between an activity and the output it led to.

### Coding and building of the meta-map

**Fig. 1:**
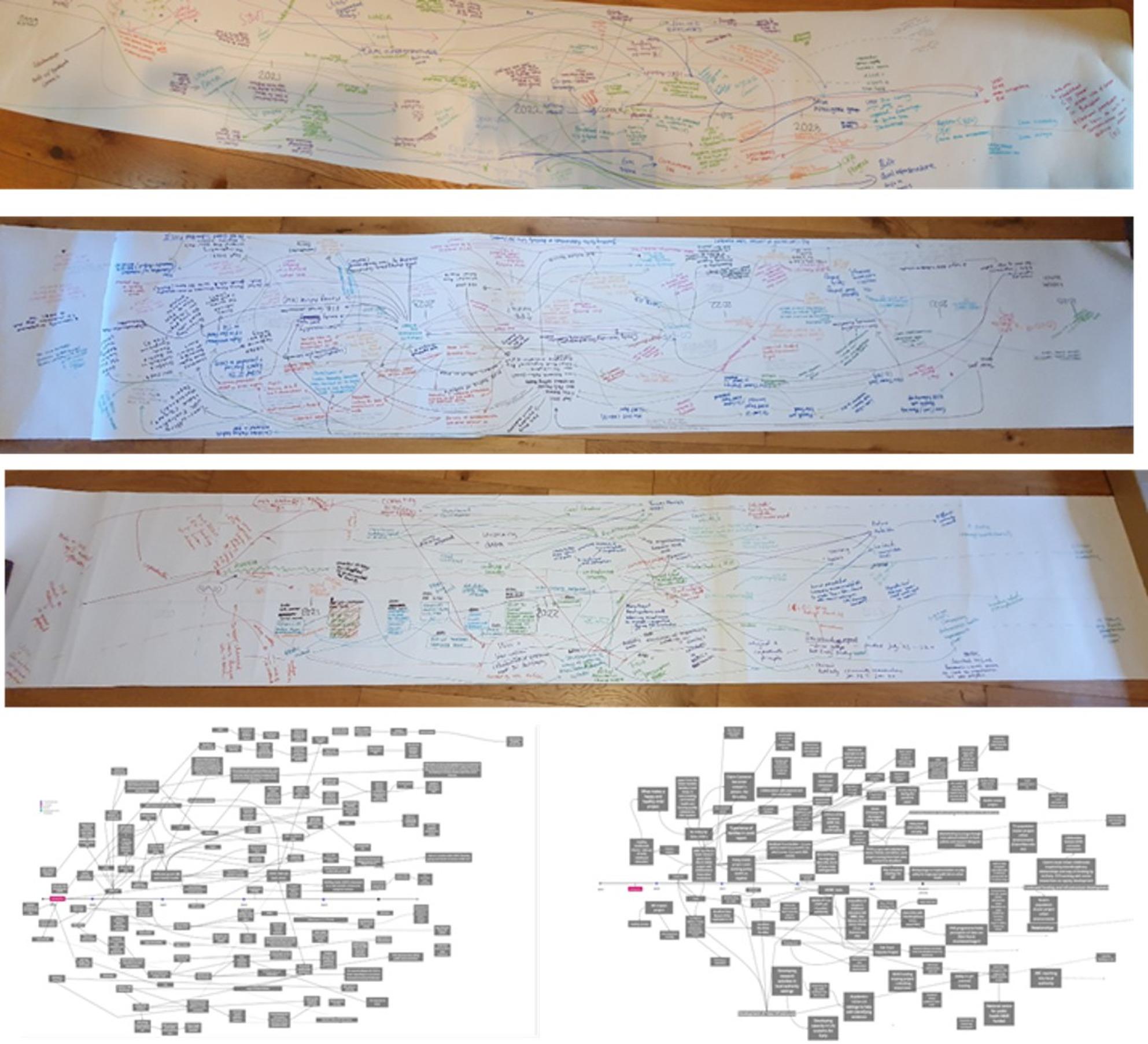
Individual REM workshop outputs

All nodes that could be allocated a date were inputted along with their allocated date into a Microsoft Excel spreadsheet using a template that is normally used for generating a network map that would build the overall meta-map. An example of the template can be found on the Open Science Framework: https://osf.io/g4jf7/overview. Nodes were then coded deductively within the spreadsheet according to the ActEarly logic model [13]. This involved each node being coded according to whether it was an input, activity, output, outcome or impact and also according to the groups of activities within the logic model (i.e., whether it related to research capacity, knowledge and evidence building, coproduction and citizen science, data infrastructure or wider system context). Each node was inputted into the Excel spreadsheet and coded once regardless of the number of REM outputs it was included in. This allowed for individual nodes included within ripples from multiple outputs to sync up when merged. A separate spreadsheet within the same workbook was then used to record which nodes followed which based on the original outputs drawing and the information included on the first spreadsheet. Activities were inputted in the order they appear within the ripples within the original REM outputs (i.e., activity B follows from activity A). This spreadsheet was then used within the software Graphiviz to create visuals of the combined ripples against the timeline of the project to depict a meta-REM map. Software previously developed for the translation and import of structured data from the spreadsheet into a graph database was extended to allow the production of GraphViz dot files that are suitable for the visualisation of ripples. Graphviz can produce ranked graphs where nodes line up with each other. This allows the nodes of the ripple effects to line up with the dates of the events. Similar visualisations can be produced using Javascript libraries, and we used d3js to produce interactive visualisations that closely match the output from Graphviz. These can be found, along with information on how to use the software to develop a meta-REM at https://github.com/prgarnett. These visualisations are more flexible allowing for highlighting key elements of the map (e.g. according to elements of the logic model) through changing the shape or colour of nodes. Fig. 1 shows individual REM outputs and final meta-REM map to visualise how multiple individual outputs can be presented in one map. The full meta-REM map can also be viewed on Open Science Framework.

### Interpretation

The total number of activities per element of the logic model were recorded to identify where most activity occurred and if this changed over the timeline of the project. The meta-REM map was also used to identify the activities that interlinked with the most ripples to report on the most influential activities within ActEarly and to explore how activities led to multiple outcomes (now more identifiable due to the merging of outputs) or had an impact across both research sites. The final meta-REM map was used to present the overall activity and outcomes of the ActEarly project. The final map and individual ripples were made public on the Open Science Framework to allow readers to explore the map in more detail and see all ripples contributing to addressing each of the research questions. Further network analysis steps can be taken here if suited to the needs of the research question.

## Results

The additional data processing and analysis steps that were taken using our new meta-REM method has provided additional analytical insights. The merging of nodes from across all outputs allows the quantification of elements of the REM. For example within the ActEarly case study it enabled reporting of the number of nodes across all outputs (*N* = 440), the number of duplicate nodes (*N* = 74) and the number of conceptual nodes that were not related to an event (*N* = 54). Data triangulation through the additional step of data verification enabled 173 nodes to be allocated a verified date; however, 63 nodes could not be dated through data triangulation and a further 76 nodes could not be included in the meta-REM map due to being linked within a ripple to a node that could not be dated. This contrasts with all 440 nodes that would have been identified and considered within the analysis following the original REM methods [1].

### Presenting findings from a meta-REM map

**Fig. 2:**
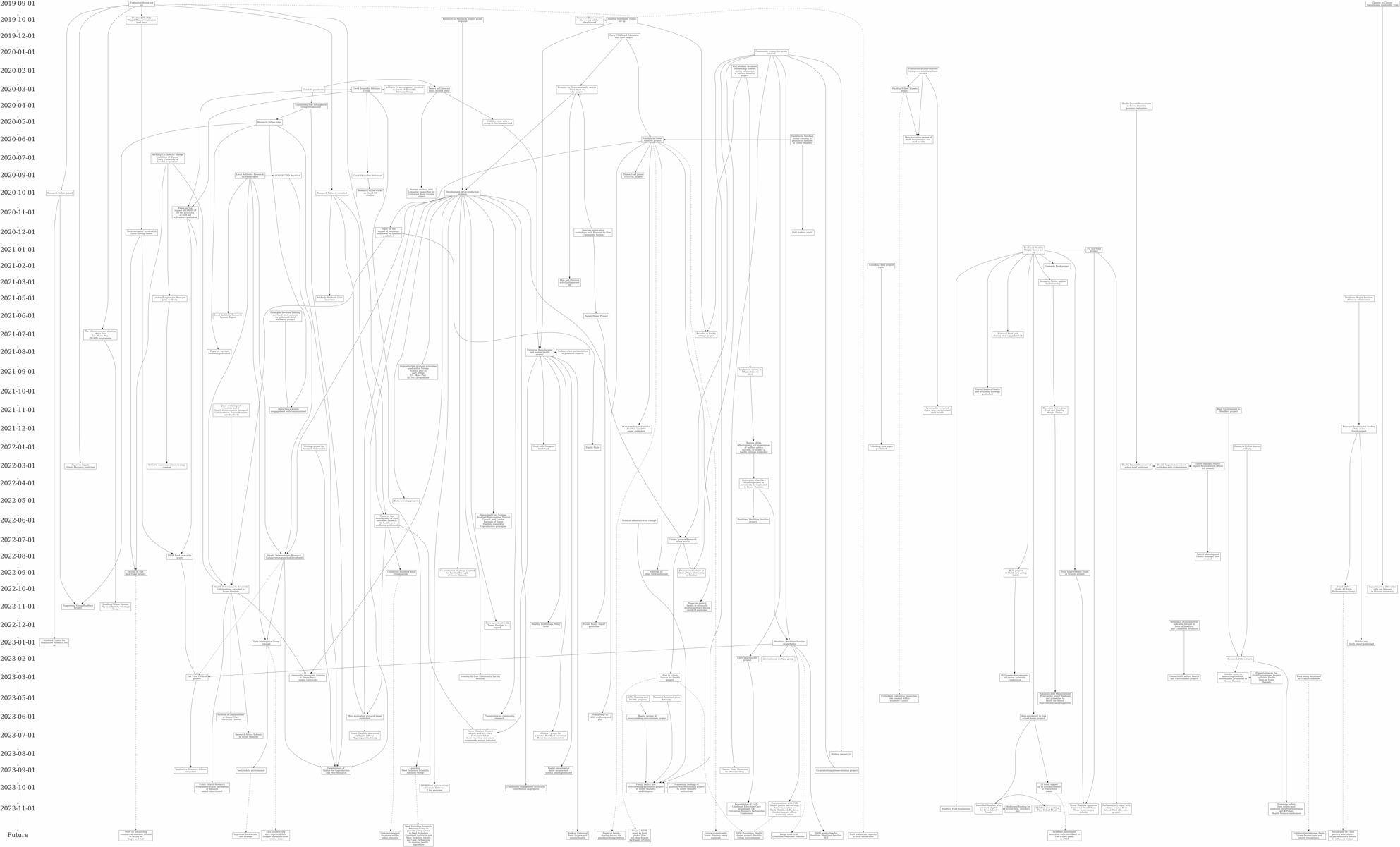
Merged Meta-REM map

The merging of REM workshop outputs and creation of a meta-REM map enabled the visualisation of the overall activities and impact of the programme. Figs. 1 and 2 show the individual REM workshop outputs and the merged meta-REM map. The meta-REM map can be viewed in more detail on the Open Science Framework ( https://osf.io/g4jf7/overview). In our ActEarly example, all nodes were coded according to the ActEarly logic model [13] which, through the building of the meta-REM map, enabled us to identify where most activity within the logic model occurred. This process contributed to the evaluation of the ActEarly programme through determining that nodes most commonly related to ‘knowledge and evidence’ (i.e., the delivery and evaluation of interventions) (*n* = 114) and ‘building research capacity’ (i.e., collaborative research groups) (*n* = 31) which were often categorised as ‘activities’ (*n* = 71) or outputs (*n* = 63). As the meta-REM map also included the programme timeline, the change in number and type of activities over the period of the programme could be visualised. Further, building an overall map shows how the number of ‘outputs’ increased over the lifetime of the programme, while the number of ‘activities’ was consistent across all years.

The creation of an overall map also enabled the identification of key events that were involved in multiple ripples. Therefore, the meta-map contributed to the ActEarly evaluation through identifying the nodes on the meta-REM map that were involved in the most ripples. This included three projects (Universal Basic Income and mental health project, Healthier Wealthier Families project pilot and Families in Tower Hamlets project), two ActEarly theme research groups (Evaluation theme and Food and Healthy Weight theme), the Health Determinant Research Collaborations in both Bradford and LBTH, the development of the ‘Nothing about us, Without Us’ coproduction strategy [15], and the creation of community engagement researcher posts.

The meta-REM map also identified where ripples included in one REM output were expanded (i.e., to include more chains of events leading from the same activity) by being linked to a ripple from another output. This shows how participants viewed that the same event led to different pathways of impact, or how multiple pathways led to the same event. REM is also able to showcase both the intended and unintended consequences of a project or programme of work [1]; therefore, the merging of outputs provided an opportunity to explore how different participants in our process were impacted by the same event in the different ways. For example, within the ActEarly case study, while in one REM output the Covid-19 pandemic was reported to have led to studies related to the pandemic being delivered (Ripple 1), within another output, it was reported that the pandemic led to delays in projects being delivered. In turn, this resulted in collaborations with other universities being formed (Ripple 19).

The expanded ripples also highlighted, where an event led to impacts across the two different research sites. For example, within the ActEarly evaluation, the development of a coproduction strategy that supported the Bradford Health Determinants Award (Ripple 5) also fed into community researchers contributing to projects in LBTH (Ripple 10). The meta-REM map also identified how the role of community researchers was included in multiple outputs in relation to their involvement in multiple projects (Ripple 27). Individual ripples can be viewed on the Open Science Framework (https://osf.io/g4jf7/overview). The meta-REM map can therefore identify how the same events can lead to wider impacts across the system (due to ripples from individual outputs being combined).

## Discussion

This paper has introduced an alternative method for handling and analysing REM data that can support the merging of multiple REM outputs to present and aid understanding of a programme’s overall pathways to impact across a system. We call this the meta-REM method that builds on and extends previous methods suggested by Chadon et al. 2017 [1] and Nobles et al., [2]. These processes may be beneficial for researchers conducting multiple REM workshops with different participant groups to consolidate their findings, those evaluating an intervention across different areas or across a system, those conducting REM across a large programme of activities and those interested in visualising the change in activities over a programme’s lifetime.

While the inclusion of a timeline was introduced by Nobles et al., [2], and was incorporated to the current evaluation of the ActEarly programme, we included an additional step of verifying dates of each node on the meta-REM map. While feedback loops (i.e., where effects feed back into a cause) are possible within complex systems [16], this method prioritises the use of the timeline and synchronising activities from multiple outputs based on dates occurring linearly. This process was necessary so that ripples could be merged, but also increased confidence in the findings of the REM by confirming the order of activities within each ripple (i.e., that each activity could have logically followed on from the previous activity based on the dates they occurred). However, the potential to include causal loops within the meta-REM visualisation could be a future area to explore. The ripples could then be used with more certainty to address the research questions within the meta-evaluation as examples of how ActEarly led to impact.

While the inclusion of ripples with verified dates increased confidence in the findings, the processes also led to the exclusion of activities that could not be dated. The verification of elements of the meta-REM map introduces quantitative elements to a method which was originally described as a qualitative method [1]. This can raise epistemological, methodological and practical considerations. Firstly, this method prioritises verification of activity dates which can reduce the inclusion of ‘perceived impact’ creating a tension between “truth” and “perception”. In taking this approach, we reflected on the often-binary categorisations of qualitative and quantitative methods. We propose that methods that do not allow for easy classification can still be very useful when applied transparently and with a focus on the research objectives. Care needs to be taken not to oversell the method, (i.e., make causal claims based on REM). It is important to decide whether it is more important to report on everything included in the workshops regardless of whether it can be verified (i.e., if participant perceptions are of most importance as within a constructivist approach) or whether providing an account of what can be proven to have ‘actually’ happened is more suited to addressing the research objectives. Secondly, practical recommendations around how feasible it is to verify the dates of activities are important to consider, and in future programmes, need to be adequately resourced in the planning stage. In addition, verifying data can require significant time and human resources which would need to be factored into the timeline and budget of the evaluation. This is particularly relevant to consider within evaluations where REM workshops are only conducted towards the end of the programme where participants are less likely to recall specific dates. Additionally the size of the programme being evaluated and the anticipated amount of data to be verified can determine the ability to verify dates.

As found within the ActEarly meta-evaluation, a large number of activities could not be dated, which reduced the total number of nodes and ripples included in the Meta-REM. This can be a limitation of the approach, particularly where the loss of one node can lead to multiple linked nodes being lost. The number of lost nodes could be partly mitigated by collecting more information and verifying dates with participants during the workshops; however it would require additional time within workshops which can already be time intensive [3]. Alternatively, utilising wider data sources (e.g., programme documentation) or advising REM workshop participants that further information would need to be collected, could increase likelihood of further engagement from participants and more data likely to be verified. A further, consideration could be to estimate dates of activities or to visualise unverified activities differently within the meta-REM map (however this was not possible within the case study due to the software requiring a date that fitted the linear timeline).

The merging of REM outputs can be beneficial for both analysing and interpreting data and presenting and disseminating findings of an evaluation across a large system of activity. Further, while this method draws on network analysis processes (i.e. how data is prepared and the software used), a full network analysis was not conducted within this case study. Further research should explore how REM data could be used within a network analysis. Through creating a meta-REM map, it was possible to identify where the same event could lead to multiple outcomes (or impacts), creating an understanding of key activities within the programme that and how it achieved impact. This can allow for more specific conclusions around the key influential activities that can contribute to successful outcomes. The merging of outputs and therefore, ripples, can also provide insight into how the same activities can impact different elements of the system, which may not have been identified within individual maps. This is particularly important when the intervention or programme is evaluated from a system perspective, as complex interdependencies and feedback loops are expected [17]. While creating a meta-REM map can be a useful tool for analysis, larger meta-REM maps may be challenging to present as a final output and the extraction of elements of the map or specific ripples can be better suited to disseminating findings. This suggests that in both larger and smaller projects, the benefit of the meta-REM may be to the researcher in aiding analysis and interpretation (i.e., through identifying merged ripples and visualising the size of activity over time). However, using the meta-REM maps to disseminate findings may be more beneficial in smaller projects.

Presenting all REM outputs against one timeline also enabled a visualisation and understanding of how the number and types of activities in the programmes changed over the lifetime of the programme. The meta-REM map can evidence if and how a programme may have grown over time and can be adapted to present key ripples or activities of interest. Different elements of the map can also be highlighted dependent on the purpose of the evaluation, such as using colours and shapes to depict different elements, or pathways through, the ActEarly logic model. The meta-REM map visualisation can also help within the presentation or dissemination of a programme’s findings and impact, and can be a way of engaging stakeholders by providing a summary visualisation of the activities, outputs and impacts of an intervention, programme or programme of work. However, the extent to which these numbers are reflective of what happened in practice is limited by what was recalled by workshop participants. This is firstly due to REM outputs only including what was discussed or was deemed important enough by the specific participants included in workshops to be reported and therefore they do not present everything that happened. This limitation has also been acknowledged by other REM methodologists [2]. Secondly, activities that could not be dated were not included in the full map.

Finally, merging REM outputs can have practical benefits in allowing an evaluation to include multiple workshops, whilst also being able to bring all findings together and presented as a whole. This could allow workshops to include fewer participants to be included within each workshop, facilitating more in-depth information to be collected (and thus, overcoming challenges of time keeping within REM workshops) [18]. Additionally, the ability to conduct multiple workshops with a combined output can overcome challenges around power imbalances and support equal participant contributions [3, 18] (e.g., through delivering smaller workshops or including specific participant groups).

## Conclusion

This paper has contributed to the advancements of the REM method and proposes optional additional steps that can aid in evaluating complex interventions and large programmes of work. The paper has presented the methods for merging and presenting REM outputs which depict reported activities and impacts across multiple REM workshops. The additional processes to REM methods outlined in this paper highlight the potential benefits to data collection (e.g., through making it feasible to conduct multiple workshops whilst still being able to present findings as whole), data analysis (e.g., through being able to interpret REM outputs as whole and see patterns of activities across the system and timeline) and to dissemination of findings through their consolidation in one meta-REM map output. These benefits are particularly relevant to those interested in exploring impacts of interventions across multiple sites, and those conducting REM with a large number of varied stakeholders. Evaluation and reflection on the use of these additional REM processes are recommended within future evaluations using REM methods.

## Supplementary Information

Supplementary Material 1.

## Data Availability

The dataset supporting the conclusions of this article is available in the Open Science Framework repository, (https://osf.io/g4jf7/overview).
